# Vitamin D supplementation to palliative cancer patients shows positive effects on pain and infections—Results from a matched case-control study

**DOI:** 10.1371/journal.pone.0184208

**Published:** 2017-08-31

**Authors:** Maria Helde-Frankling, Jonas Höijer, Jenny Bergqvist, Linda Björkhem-Bergman

**Affiliations:** 1 ASIH Stockholm Södra, Långbro Park, Palliative Home Care and Hospice Ward, Älvsjö, Sweden; 2 Department of Laboratory Medicine, Division of Clinical Microbiology, Karolinska Institutet and Karolinska University Hospital, Huddinge, Stockholm, Sweden; 3 Unit of Biostatistics, Institute of Environmental Medicine, Karolinska Institutet, Stockholm, Sweden; 4 Department of Surgery, Breast Centre, Capio St Gorans Hospital, Stockholm, Sweden; 5 Department of Oncology-Pathology, Karolinska Institutet, Stockholm, Sweden; Roswell Park Cancer Institute, UNITED STATES

## Abstract

**Background:**

We previously showed an association between low vitamin D levels and high opioid doses to alleviate pain in palliative cancer patients. The aim of this case-controlled study was to investigate if vitamin D supplementation could improve pain management, quality of life (QoL) and decrease infections in palliative cancer patients.

**Methods:**

Thirty-nine palliative cancer patients with levels of 25-hydroxyvitamin D < 75 nmol/L were supplemented with vitamin D 4000 IE/day, and were compared to 39 untreated, matched “control”-patients from a previous study at the same ward. Opioid doses, antibiotic consumption and QoL-scores measured with the Edmonton Symptom Assessment Scale (ESAS) were monitored. The primary endpoint was the change from baseline after 1 and 3 months compared between the groups using linear regression with adjustment for a potential cofounding factor.

**Results:**

After 1 month the vitamin D treated group had a significantly decreased fentanyl dose compared to the untreated group with a difference of 46 μg/h; 95% CI 24–78, which increased further at 3 months to 91 μg/h; 95% CI 56–140 μg/h. The ESAS QoL-score improved in the Vitamin D group the first month; -1.4; 95% CI -2.6 - (-0.21). The vitamin D-treated group had significantly lower consumption of antibiotics after 3 months compared to the untreated group, the difference was -26%; 95%CI -0.41%–(-0.12%). Vitamin D was well tolerated by all patients and no adverse events were reported.

**Conclusion:**

Vitamin D supplementation to palliative cancer patients is safe and improvement in pain management is noted as early as 1 month after treatment. Decreased infections are noted 3 months after vitamin D treatment. The results from this pilot-study have been used for the power-calculation of a future randomized, placebo-controlled, double-blind study called “Palliative-D” that will start in Nov 2017 and will include 254 palliative cancer patients.

## Introduction

Palliative cancer patients often suffer from pain and infections, which may reduce their quality of life and shorten their remaining life span. In palliative medicine, good symptom management and prevention of new symptoms are major priorities, aiming to maintain or even improve the patient’s quality of life. Since pharmacological treatment often has unwanted side effects, the palliative caregiver faces the challenge of treating disease related symptoms without causing harm.

Vitamin D is synthesized in the skin using energy from UVB-light. Vitamin D is further hydroxylated in two steps into the active form 1,25-dihydroxyvitamin D, which binds to the vitamin D receptor (VDR). The activated VDR complex regulates a large number of genes [1] and vitamin D is important for a healthy immune system since it induces the synthesis of antimicrobial peptides in immune cells and on mucosal surfaces as a part of the “first line defense” against invading microbes [2].

The pro-form 25-hydroxyvitamin D (25-OHD) is more stable than the active 1,25 hydroxyvitamin D, with a half-life of about 3 weeks compared to 4 hours [3] which is why it is used for evaluation of vitamin D status [4]. Serum levels of 25-OHD below 50 nmol/L are considered to be insufficient according to the Institute of Medicine in the US [5]. Furthermore, there is increasing evidence that levels of 25-OHD above 75 nmol/L are optimal for the immune system [6,7].

Low serum levels of vitamin D are more common in palliative cancer patients than in healthy controls [8–10]. Both observational and interventional studies suggest a role of vitamin D in pain intensity and in management of pain in varying clinical settings [8,11–16]. Nevertheless, 3 meta-analyses reviewing previously performed randomized, controlled trials (RCTs) could not establish a correlation between vitamin D supplementation and pain reduction [14,17,18]. In contrast, a recent review of published RCTs concluded that a significantly greater mean decrease in pain score could be observed with vitamin D supplementation compared to placebo treatment in patients with chronic pain [19]. There is also increasing evidence that vitamin D supplementation may reduce depressive symptoms and improve quality of life [20,21].

A previous prospective, observational study performed at our palliative care unit in Stockholm, studied the relationship between 25-OHD levels and opioid dose, infections and quality of life [8]. In this study, lower serum levels of 25-OHD correlated with a higher fentanyl dose. However, there was no correlation between serum 25-OHD levels and infections or quality of life. No vitamin D-supplementation was given in that study. After this study we have changed the guidelines at our ward and patients with 25-hydroxyvitamn D levels of < 75 nmol/L are now offered vitamin D supplementation. In the current study we wanted to determine if the vitamin D supplementation could improve pain management, reduce infections and improve quality of life in our palliative cancer patients. Finally, we also wanted to evaluate if there were any negative side effects of the treatment in this patient group.

## Methods

### Study cohort

This is a case-control study where the vitamin D supplemented patients were followed prospectively with regards to their opioid consumption, infections and quality of life. The supplemented patients were later matched with untreated control-patients from a previous study at the same ward, performed before the new guidelines regarding vitamin D-treatment were introduced. In this study cohort, palliative cancer patients were supplemented with vitamin D 4000 IE/day (Detremin) and followed for up to 3 months.

Palliative cancer patients were recruited consecutively when they were admitted to ASIH Stockholm Södra, Långbro Park Advanced Palliative Home Care Team or Hospice Ward from Sept 2015 to June 2016. Inclusion criteria were: age 18 or above, incurable cancer (any type of cancer), expected survival time of more than 1 month and serum levels of 25-OHD < 75 nmol/L, i.e. “insufficient levels” according to local guidelines at Karolinska University Hospital Stockholm. The patients were supplemented with vitamin D3 (Detremin: cholecalciferol (vitamin D3) dissolved in Miglyoil). Patients took 8 drops of this Detremin every day; corresponding to 4000 IE = 100 μg. All patients that survived for more than 1 month were included in the final analysis.

### Control group

Each treated patient was matched with an untreated control. Controls were taken from a previous observational vitamin D study at our ward (n = 169) that was started 2014. The first 100 patients included in that study are described in a previous publication [8]. In that study the same parameters as in the present study were monitored, and 25-OHD was measured, but no vitamin D treatment was given. The patients in the study cohort were matched with untreated controls regarding, sex, approximate age (±10 years), cancer type (divided into the 8 groups: breast cancer, GI-cancer, lung cancer, pancreatic cancer, prostate cancer, gynecological cancers, cholangiocarcinoma and head and neck cancer), 25-OHD level at baseline, survival time (1–3 month, 3–6 months or more than 6 months after inclusion) and number of days in the study. To the greatest possible extent, the matched treated and untreated patients had the same cancer type, but in the GI-cancer group a treated patient with small bowel cancer was matched with an untreated patient with colon cancer, and one patient in the intervention group with rectal cancer was matched with a control with esophageal cancer.

### Data extraction

Patients with detected vitamin D deficiency, i.e. 25-OHD levels < 75 nmol/L according to local guidelines, were asked to participate in the study. After inclusion the demographic data regarding age, sex and type of cancer were retrieved from the patients’ medical records and levels of 25-OHD, CRP and albumin at baseline were measured. The levels of 25-OHD in serum were analyzed by chemiluminescence immunoassay (CLIA) on a LIAISON-instrument (DiaSorin Inc, Stillwater, MN, USA,) with a detectable range of 7.5–175 nmol/L, CV 2–5% at the Department of Clinical Chemistry, Karolinska University Hospital. Opioid dose, infections and ESAS QoL-score were recorded at baseline, and after 1 month and 3 months (for surviving patients). After 3 months the 25-OHD levels, CRP, albumin and S-calcium were monitored again. Survival time was recorded.

Infections were defined as the percentage of days with antibiotic treatment the month before study inclusion. Opioid dose at the day of inclusion was recorded and translated to the corresponding fentanyl dose (μg/ hour). Self-assessed QoL was recorded with the Edmonton Symptom Assessment Scale (ESAS) [22], that is routinely monitored every second week in all patients at ASIH Långbro Park. Using the ESAS scale 10 different parameters are assessed, where QoL is one such parameter. The ESAS-scale ranges from 0–10, where the number 10 is the ‘worst possible quality of life’ and 0 represents a ‘good quality of life’ [22].

Data from treated individuals with a survival time of less than 3 months were matched with data from the corresponding time before death for untreated individuals, i.e. if the individual lived for 40 days after inclusion, data from the 40 last days in life of the untreated matched control were collected.

The raw data is available in S1 Table. However, in accordance with the approved applications to the Regional Ethical Review Board we have removed the age and some other personal data of the included subjects from the dataset to make it impossible to identify single patients.

### Statistical analysis

Statistical analyses were performed using Stata v. 13.1. Baseline characteristics for the two groups were compared using paired t-test for continuous variables, McNemar's test for binary variables and Wilcoxon signed rank test for “number of days in the study”. CRP was log-transformed due to skewness, before conducting the paired t-test. The paired t-test was also used to compare vitamin D levels between baseline and after three months in the vitamin D group, among the 23 patients that have a measured vitamin D level at both time points.

The comparisons of the change from baseline in opioid dose, infections and quality of life between the two groups were made using crude fixed effects linear regression, where the fixed effects take the matched structure of the data into account. Due to heteroscedasticity and skewness in the outcome variables, bias corrected and accelerated bootstrap [23] were used to calculate 95% confidence intervals for the mean differences. Since there was an imbalance concerning CRP between the groups, an adjusted analysis was also made for each crude comparison, using a log-transformed version of CRP as covariate.

### Ethical statement

The studies were approved by the Regional Ethical Review Board, Stockholm, Sweden; Dnr: 2015/776-31 (vitamin D treatment) and 2014/455-31/4 (controls without treatment) and was performed in accordance with the declaration of Helsinki. Written informed consent was obtained from all patients, both cases and controls, before inclusion in the studies. This consent gave us the opportunity to collect data retrospectively 3 months before inclusion and up to one year after inclusion.

## Results

### Demography of cases and controls

**Fig 1:**
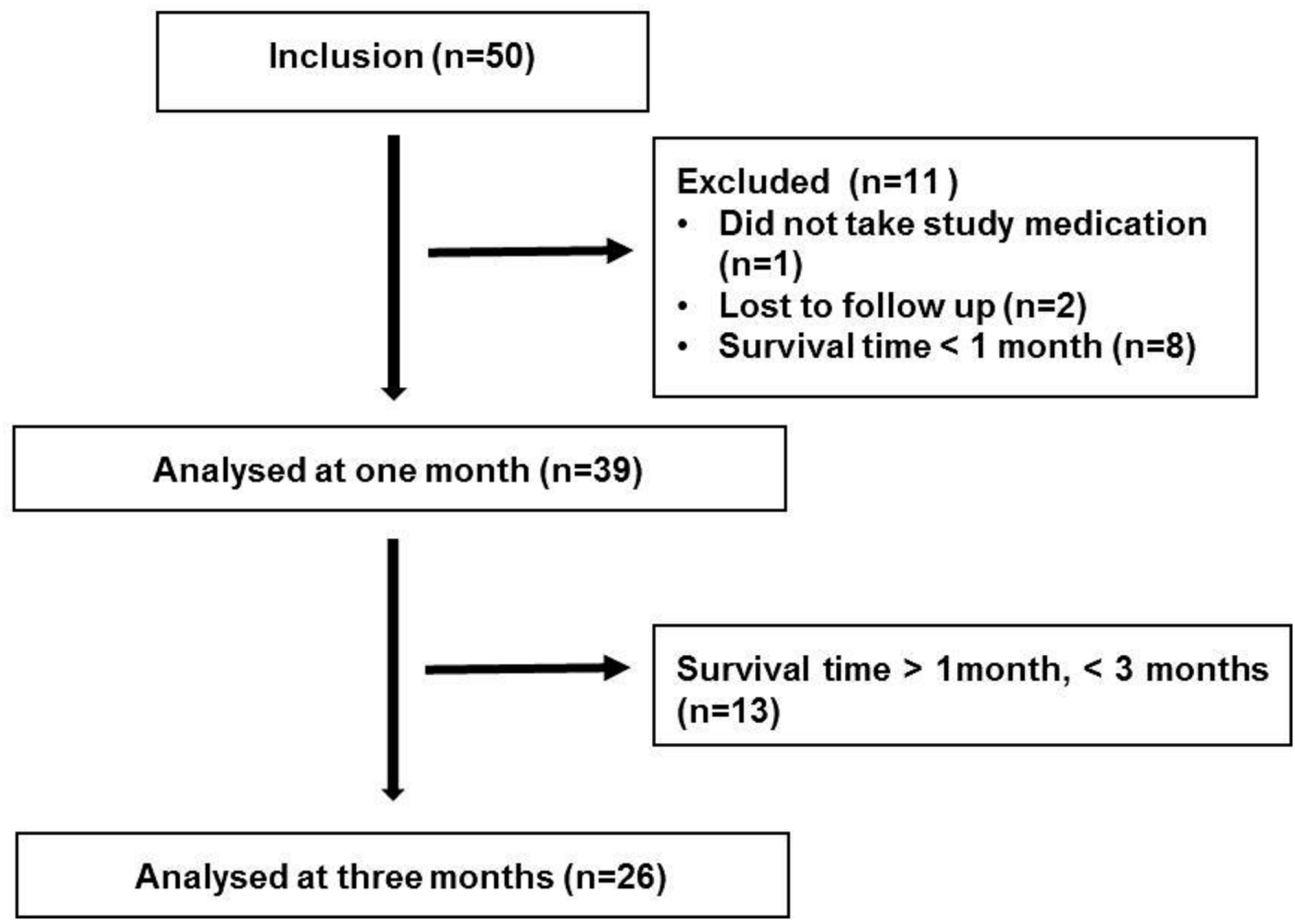
Flowchart of patients included in the vitamin D intervention study.

Fifty patients were included in the vitamin D treated group. Eight patients died within one month of treatment, one patient did not take the vitamin D oil and was excluded, and 2 patients were lost to follow up since they were dismissed from the Palliative Home Care Team. Thirty-nine patients, 18 men and 21 women, survived more than 1 month and were included in the final analysis and were thus matched with untreated patients from the previous study cohort [8]. Twenty-six patients survived more than 3 months and could be included in the 3 month follow up (Fig 1). The mean age was 62 years (SD 13). All patients included in the study were palliative cancer patients, and the diagnoses were breast cancer (n = 6), colorectal cancer (n = 11), lung cancer (n = 5), pancreatic cancer (n = 4), gynecological cancer (n = 3), cholangiocarcinoma (n = 2) and head and neck cancer (n = 5).

Fifty-six % of vitamin D treated and 49% of non-vitamin D treated patients were given palliative chemotherapy at baseline. Three of six patients with breast cancer in the intervention group and four of six breast cancer patients in the untreated cohort, as well as all patients with prostate cancer in both groups, were on anti-hormonal treatment at the time of inclusion.

### Matching

There was no statistically significant difference between the intervention group and the untreated cohort in the baseline values except for CRP and ESAS QoL-score. The demography of all patients is shown in Table 1.

**Table 1:** Baseline demography of vitamin D treated patients and untreated controls. The figures show total number and percentage or mean ± SD.

Parameter	Vitamin D (n = 39)	Controls (n = 39)	p-value
**Sex**			
Male (n)	18 (46%)	18 (46%)	
Female (n)	21 (54%)	21 (54%)	1
**Age (SD)**	61,9 ± 13	62,4± 13	0.65
**Type of cancer (n)**			
CNS	1	1	
Breast	6	6	
Colorectal	11	11	
Lung	5	5	
Gynecological	3	3	
Pancreas	4	4	
Cholangiocarcinoma	2	2	
Head-Neck	2	2	
Prostate	5	5	
**Number of days in the study (SD)**	76 ± 21	76 ± 21	1
**Survival > 90 days** (n)	26	26	1
**Vitamin D** (nmol/L)	33 ± 26	38 ± 18	0.34
**CRP** (mg/L)	47 ± 76	67 ± 75	0.01*
**Albumin** (g/L)	28 ± 6	27 ± 7	0.15
**ESAS QoL** (points)	5,49 ± 2,0	4,05 ± 2,4	0.02*
**Infections** (days of antibiotics)	16% ± 19%	15% ± 27%	0.83
**Pain** (μg fentanyl/h)	31 ± 48	43 ± 5	0.20
**Chemotherapy** (n)	22	19	0.51

There was no statistically significant difference between treated and untreated patients except for CRP and ESAS QoL-score:

*p<0.05.

As stated in the statistical analysis section, there was an imbalance concerning CRP between the treatment group and the controls. The adjusted analysis made for each crude comparison yielded no significant difference from the unadjusted analysis, and in the following text the unadjusted data is presented. Both adjusted and unadjusted data are presented in Tables 2 and 3.

**Table 2:** Results from crude and adjusted analysis of change in fentanyl dose, ESAS QoL-score and infections (% days with antibiotics) compared to baseline in vitamin D treated and untreated patient after 1 month. Figures show mean value (95% CI).

1 month (n = 39)				
Outcome		Mean change from baseline	Crude mean difference in change	Adjusted mean difference in change
**Fentanyl**	**Treated**	6 μg/h	-46 μg/h (-78 –(-24)	-60 μg/h (-101– (-28))
	**Untreated**	52 μg/h		
**ESAS**	**Treated**	-1.1	-1.4 (-2.6 –(-0.21))	-1.4 (-2.90 –(-0.60))
	**Untreated**	0.2		
**Infections**	**Treated**	-6%	-9% (-20%– 2%)	-6% (-17% –8%)
	**Untreated**	3%		

**Table 3:** Results from crude and adjusted analysis of change in fentanyl dose, ESAS QoL-score and infections (% days with antibiotics) compared to baseline in vitamin D treated and untreated patient after 3 month. Figures show mean value (95% CI).

3 months (n = 26)				
Outcome		Mean change from baseline	Crude mean difference in change (95% CI)	Adjusted mean difference in change (95% CI)
**Fentanyl**	**Treated**	-6 μg/h	-91 μg/h (-140 –(-56))	-120 μg/h (-185 –(-49))
	**Untreated**	85 μg/h		
**ESAS**	**Treated**	-1.3	-1.6 (-3.1–0)	-2.0 (-4.4–0.72)
	**Untreated**	0.3		
**Infections**	**Treated**	-13%	-26% (-41%–(-12%))	-29% (-49%–(-11%))
	**Untreated**	13%		

### Opioid dose

At baseline there was no statistically significant difference in opioid doses between the groups. The mean fentanyl dose in the vitamin D treated patients was 31 μg/h at inclusion (n = 39), 37μg/h after one month (n = 39) and 22 μg/h after three months (n = 26). In the control group the mean fentanyl dose increased from 43 μg/h at baseline (n = 39), to 95 μg/h after one month (n = 39), and 117 μg/h (n = 26) after three months observation time.

**Fig 2:**
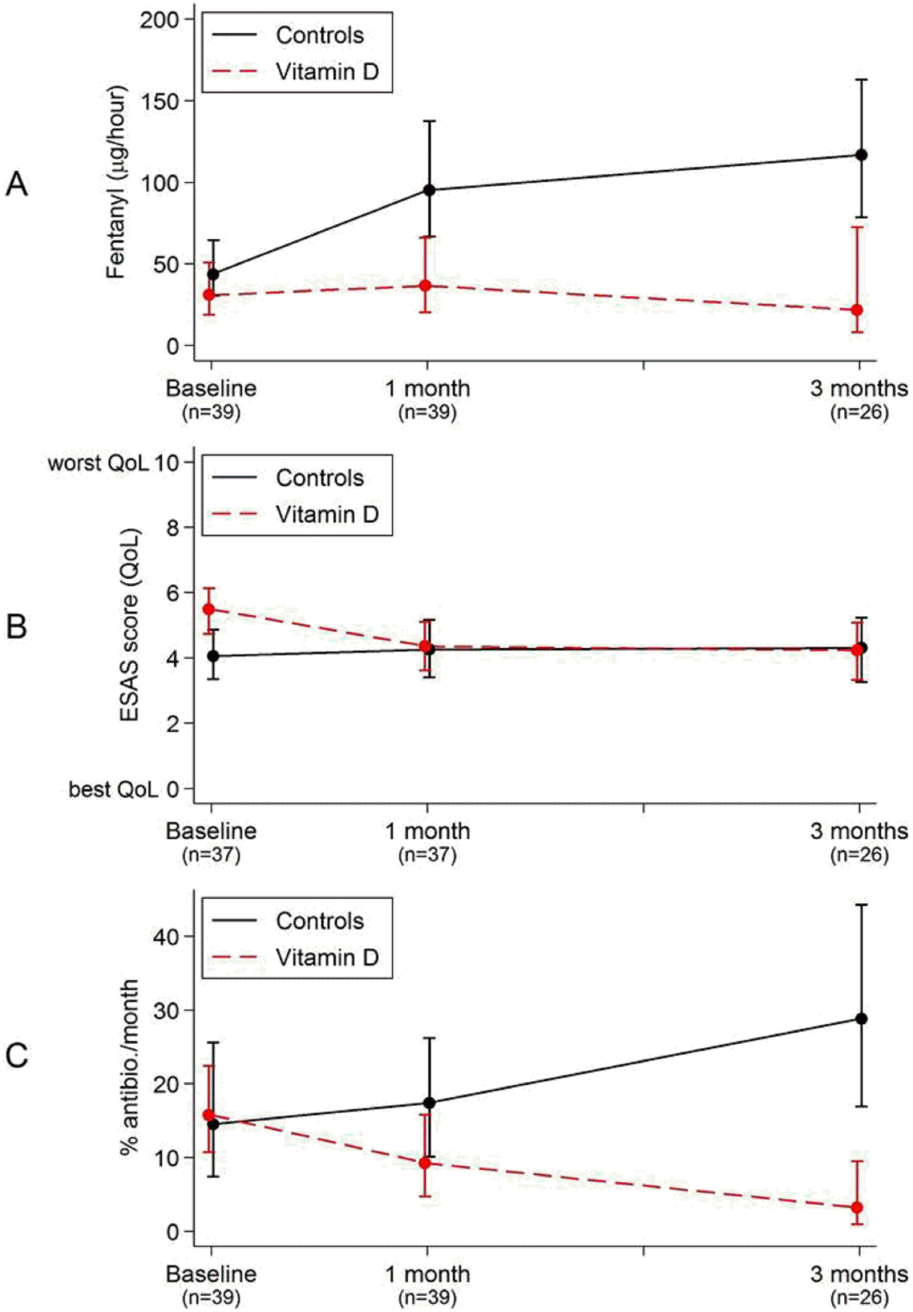
Differences in opioid dose (μg fentanyl/h) (A), Quality of Life (according to ESAS-assessment) (B) and antibiotic consumption (% of days with antibiotic the month before) (C) between 39 vitamin D treated palliative cancer patients and 39 matched controls. Points show mean values and bars show 95%CI. There was a significant difference in fentanyl dose between the groups as soon as 1 month after treatment and in antibiotic consumptions after 3 months.

The change in fentanyl dose between the groups was analyzed by linear regression with bias corrected and accelerated bootstrap and revealed a statistically significant difference already after 1 month; 46 μg/h (95% CI 24–78 μg/h), which increased even further at three months; 91 μg/h (95% CI 56–140 μg/h) (Tables 2 and 3, Fig 2A).

In the treatment group 36% of patients (n = 14) reduced their daily opioid dose during the follow up period. Out of these 14 patients 8 patients were on active palliative oncological treatment (chemotherapy and/or hormonal therapy) at the time of inclusion. In the control group we observed reduction of opioid dose in one patient over time. This patient was treated with palliative chemotherapy at baseline.

In the treatment group 28% of patients (n = 11) were not treated with opioids at baseline, and these patients remained opioid free at one month (n = 11) and at three months (n = 7). In the treatment group 18% of patients (n = 7) stopped taking opioids during the study period (fentanyl dose at baseline 12–25 μg/h). In the control group, 12 patients were not on opioids at baseline. Six patients in the control group were still opioid free after one month, and five patients were opioid free after three months.

### Quality of life

At baseline there was a significant difference in the mean ESAS QoL-score between the vitamin D treated group and the controls. The mean ESAS QoL-score was 5.5 in the vitamin D group and 4.1 in the control group, i.e. the vitamin D group had lower self-assessed QoL compared to the control group. A small improvement in the ESAS QoL-score (i.e. decrease in ESAS-score) among the vitamin D treated patients was observed after one month, eliminating the difference between the two groups; -1.4 (95% CI -2.6-(-0.21) (Tables 2 and 3, Fig 2B). After three months no further significant changes in either group were seen.

### Infections

One month after treatment with vitamin D there was a trend towards lower incidence of infections in the vitamin D treated group compared with the matched, untreated controls. This was due to an increased number of days on antibiotic treatment in the untreated group, and a reduction of the number of days on antibiotics in the vitamin D treated cohort. After 3 months of vitamin D supplementation a statistically significant mean difference between the groups with regards to infections could be observed, with lower values in the vitamin D treated group; -26% (95% CI -41%–(-12%)) (Tables 2 and 3, Fig 2C).

### Compliance

**Fig 3:**
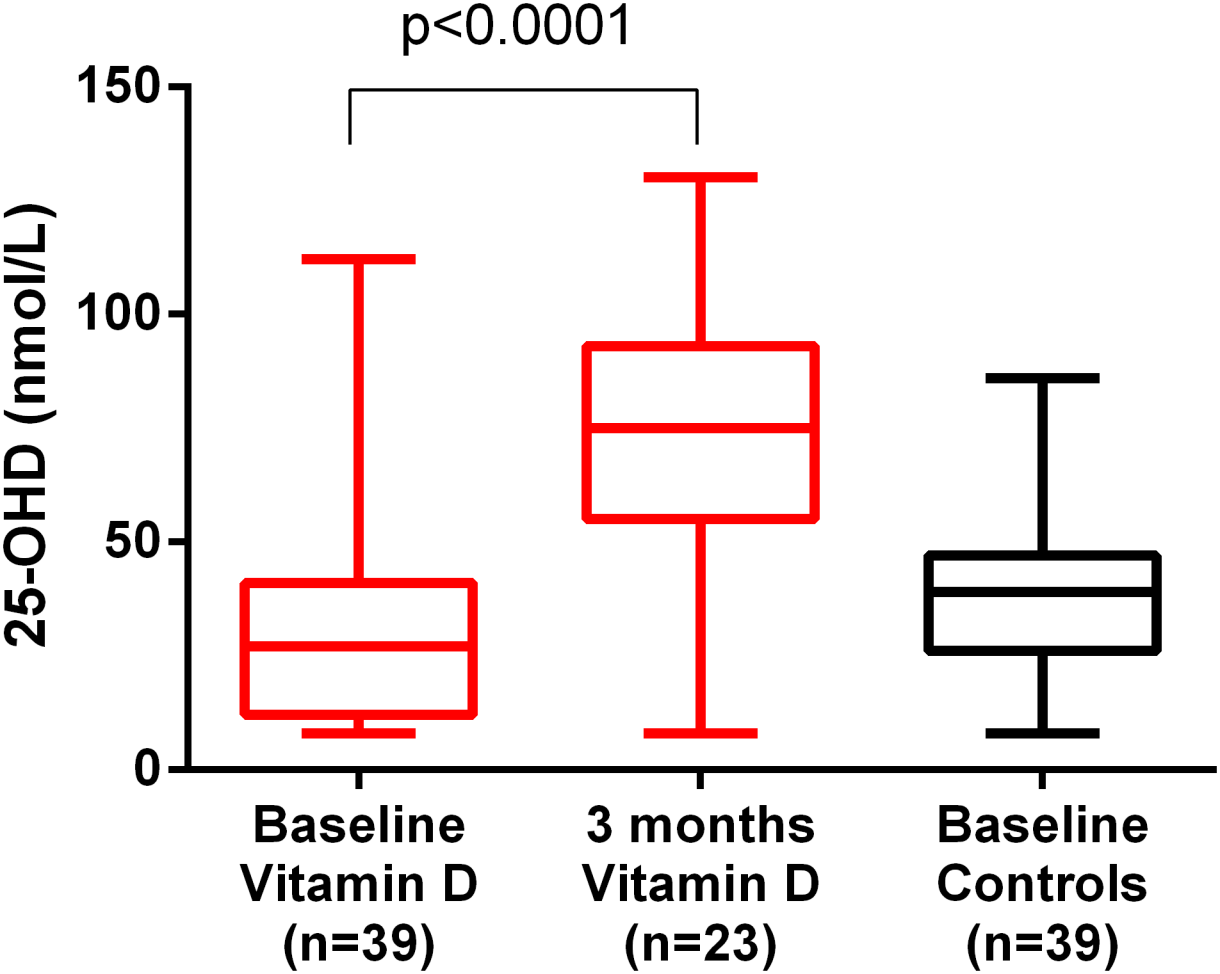
25-hydroxyvitamin D (25-OHD) levels in the vitamin D (n = 39 and n = 23) and control group (n = 39). There was a significant increase of 25-OHD levels in the vitamin D group compared to baseline; statistical analysis was performed by using a paired t-test (p<0.0001). There was no statistically significant difference in baseline values between the vitamin D and control group. There were no follow-up measurements performed in the control group. The lines show median value, the boxes 25–75 percentile and whiskers show minimum and maximum.

Follow-up monitoring of 25-OHD was performed after 3 months in 23 patients and showed a mean value of 73 nmol/L (SD 31) which was a significant increase from baseline as shown in Fig 3 (p<0.0001). Only two patients did not have increased 25-hydroxyvitamin D levels. One of these patients, a man with GI-cancer and inflammatory bowel disease, had undetectable levels (< 8 nmol/L) both at baseline, and after 1 month and 3 months. However, we perceived him as meticulous about his medication. He also asked for a new bottle after 1.5 months and wanted follow-up monitoring regarding serum levels of 25-OHD already after 1 month. He had a very short small bowel (less than 0.5 m) and as such it was likely that he did not have sufficient absorption of the vitamin D. This patient died less than 4 month after inclusion. He had a small improvement in pain-management the first month after inclusion, but no significant change in QoL or antibiotic consumption. Another vitamin D-treated patient, a woman with GI-cancer, had a decrease in serum levels of 25-OHD from 62 nmol/L to 32 nmol/L after 3 months. In her medical records, there is no information on compliance. However, a new bottle of Detremin was given to her after 2 months. She had unchanged parameters throughout the study on pain, QoL and antibiotic consumption.

The levels of 25-OHD was never measured in the control group after 3 months since these patients did not receive any treatment. Thus, only the baseline levels of 25-OHD in the control group is shown in Fig 3.

### Side effects

No side effects, including hypercalcemia, were reported in the intervention group. Safety samples were collected after 3 months (for those still alive) including, 25-OHD, calcium, albumin and creatinine.

## Discussion

In this study we show that vitamin D supplementation in palliative cancer patients who had 25-OHD < 75 nmol/L resulted in decreased administration of opioids already after 1 month compared to untreated control patients. In addition, there was a significant reduction in antibiotic consumption among the vitamin D treated patients after three months of supplementation. Importantly, there were no documented side effects of vitamin D supplementation in the intervention group.

To our knowledge, no results from randomized, clinical trials on vitamin D supplementation in palliative cancer patients have been published. However, the effect of 10 000 IU D-vitamin/day, in combination with calcium, in bisphosphonate treated breast cancer patients with bone metastases was investigated where each patient was its own control. In this study, vitamin D supplementation had no effect on self-reported pain intensity, but there was a significant decrease in number of pain sites over time [24]. Previous randomized, controlled trials of vitamin D treatment of non-cancer patients with chronic pain have shown divergent results [14,17–19].

When evaluating results from clinical vitamin D trials, it is important to consider baseline levels of 25-OHD [25]. If patients with sufficient vitamin D levels at baseline are included in an intervention study, they will not benefit from treatment. This is often forgotten when vitamin D studies are evaluated and compared. In the current study we have only included patients with insufficient levels of vitamin D.

An advantage of our study compared to previous studies is that we can observe the effect on opioid doses, which is a more objective marker for pain, than for example VAS-scores, which are used more frequently in pain-studies. However, we only study opioid-dependent pain, i.e. nociceptive pain, in this study and neurological pain or other types of pain, e.g. existential pain, were not studied.

The physiological mechanism linking vitamin D to pain is not yet elucidated. Evidence from both clinical and animal studies suggest that insufficient levels of vitamin D affect both peripheral [26,27] and parasympathetic nerve function [28]. Vitamin D also has anti-inflammatory effects, especially on the T-cell response [29], and this might decrease inflammatory-mediated pain. One hypothesis is that UVB-exposure increases the production of analgesic beta-endorphins, which suggest that serum levels of vitamin D could serve as a marker for UVB exposure, and would then not directly be involved in the pain mechanism [30]. However, if this was the only explanation then vitamin D treatment would not have been successful in reducing pain in our study.

In our study the opioid doses in the untreated group continued to increase over time during the observation period of up to 90 days. This is the natural scenario in palliative cancer patients, and our results are in accordance with the results from a retrospective, population based study looking at opioid use over time in palliative cancer patients during the last 11 months of life, where daily opioid doses continued to increase in all groups until the last recorded observation two weeks before death [31].

Several observational studies have reported a correlation between vitamin D deficiency and decreased QoL in different patient-populations [32–36]. In a previous observational study at our center, no correlation was seen between low 25-OHD levels and QoL in palliative cancer patients [8]. In a cross sectional study on 30 palliative cancer patients in Spain, no statistically significant association between 25-OHD and overall QoL was observed, but higher vitamin D status correlated with absence of fatigue and improved physical and functional well-being [37].

Vitamin D plays an important role in the immune system by inducing antimicrobial peptides on mucosal surfaces and in immune cells, as well as downregulating cytokines and reducing inflammation [2,29]. In a meta-analysis on 11 randomized, controlled trials vitamin D treatment was shown to reduce the number of respiratory tract infections [38]. The meta-analysis also showed that the protective effect of vitamin D was larger in studies using once-daily dosage compared to bolus doses.

No studies so far have considered the effect of vitamin D treatment on the infections in cancer patients. In palliative cancer patients, infections as well as antibiotic treatment are frequent. In a recent retrospective study at our site, 49% of patients were treated with antibiotics during the last two weeks of life [39]. The results from our present study suggest that vitamin D supplementation in patients with low serum levels of vitamin D can reduce the number of days the patients were treated with antibiotics, thus reducing the risk for negative side effects, as well as the risk of increasing the frequency of multi-resistant bacteria. Since antibiotics towards end of life in palliative cancer patients is often administered intravenously [39], reducing the number of days on antibiotic treatment can improve the individual patient’s autonomy and facilitate home care.

Despite the potential effect on pain, infections and QoL, this study has several limitations which need to be considered. First, the case-control design has its inherent limitations since it is difficult to find representative control patients. In addition, the controls have been selected from a historic cohort from a previous study at the same ward. The difference in time between the collected data in the two groups might potentially have affected the results since it cannot be excluded that the prescribing pattern of opioids or antibiotics might have changed over the two year period between the studies. Although there were no large differences in baseline demography between the groups as presented in Table 1 it should be noted that the control-group had higher CRP levels at baseline, which might be a marker of a heavier cancer-burden in that group. In addition, there were 3 more patients on chemotherapy in the vitamin D group compared to the control-group, which might also have contributed to the reduction in pain. The small sample size of this study (39+39) is also a limitation. To confirm the findings presented here, larger, randomized trials are needed. In fact, the data from this study has been used for a power-calculation for a future double-blinded, randomized, controlled trial (n = 250) called”Palliative-D”, which is registered at Clinicaltrial.gov, and will start in September 2017 at our ward.

Another weakness of this study is the fact that the studied cohort is heterogeneous with regards to the type of tumor, ongoing oncological treatment and remaining life expectancy, making it difficult to control extraneous variables. However, in a narrative review discussing possible evidence-based approaches in palliative research, Aoun *et al* argue that findings from heterogeneous cohorts such as ours are in fact well generalizable in different palliative care settings because of the proximity to clinical reality [40].

Despite these limitations, the findings presented here are still hard to explain without assuming some effect of the supplement.

The absence of a large effect in QoL despite the positive results on pain and infections might be explained by the fact that the QoL depends on much more than merely opioid-dependent pain and infections. As such these small effects that might improve the day-to-day life for these patients, without additional side-effects, must be seen as an important contribution to the treatment of palliative cancer patients. An addition benefit of the supplement is that since Detremin is an oil-based supplement (oral solution) it is easy to swallow also for patients that have difficulties in swallowing tablets.

## Conclusion

Vitamin D treatment in palliative cancer patients may reduce opioid doses, reduce infections and improve QoL without causing harm to the patients. However, larger randomized, placebo-controlled studies are needed to confirm the results from this pilot-study before any form of conclusions can be drawn. Thus, the results from this pilot-study have been used for the power-calculation of a future randomized, placebo-controlled, double-blind study called “Palliative-D”. This study will start in Stockholm, Sweden, Nov 2017 and will include 254 palliative cancer patients from several different Palliative Home Care Teams. The results from Palliative-D will provide an increased possibility to evaluate the true effects and possible draw backs of vitamin D treatment in palliative cancer patients and will be used to develop new treatment guidelines.

## Supporting information

S1 Table Raw data from the case-control study. (PDF)
