# Supplementary material for: Vitamin D supplementation to palliative cancer patients shows positive effects on pain and infections—Results from a matched case-control study
Source: PLoS One. 2017 Aug 31;12(8):e0184208. doi: 10.1371/journal.pone.0184208 (PMC5578640; doi:10.1371/journal.pone.0184208)
Supplement: S1 Table — (PDF) [file pone.0184208.s001.pdf]

Supplementary Information

S1 Table 1

S1 Table 1

Cases (vitamin D)	Sex 1=male	25-OHD nmol/L	CRP mg/L	Albumin g/L	**QoL** ESAS-score baseline	1 month	3 month	**Infections** percentage days with antibiotics baseline	1 month	3 month	**Pain** Phentanyl/hour baseline	1 month	3 month	**survival time** >3 months	> 6 months	Cancer	ongoing chemo.	ongoing anithorm. therapy	25-OHD 3 months nmol/L
1	1	10	4	30	7	5	3	0,1	0	0	75	25	0	1	1	GI	1		78
2	1	10	9	29	nd	nd	nd	0	0	0	100	75	37	1	1	Pancreas	0		75
3	0	8	194	19	8	8	nd	0,3	0,2	nd	100	200	nd	0	0	Gyn	0		
4	0	56	8	26	2	2	7	0,33	0,07	0	0	0	0	1	0	Breast	1		105
5	0	35	245	17	8	8	nd	0,33	0,33	nd	25	0	nd	0	0	Lungcancer	0		
6	0	10	10	28	4	4	0	0	0	0	25	12	0	1	1	Pancreas	1		48
7	0	37	77	23	10	3	nd	0,23	0	nd	125	137	nd	0	0	Cholangiocarcinoma	0		
8	1	12	8	15	4	6	nd	0,33	0,3	nd	25	75	nd	0	0	Cholangiocarcinoma	0		
9	0	20	1	40	5	2	2	0	0	0	12	12	0	1	0	Head-Neck	0		56
10	0	11	15	32	7	5	5	0,42	0,4	0	12	0	0	1	0	Breast	1	1	
11	0	20	5	26	6	3	3	0	0,33	0,33	12	0	0	1	1	Breast	1		78
12	0	41	23	28	4	5	6	0	0,47	nd	25	75	nd	0	0	Lungcancer	0		
13	1	31	22	21	7	5	5	0,33	0,17	nd	0	0	nd	0	0	GI	0		
14	0	27	33	28	5	5	nd	0,21	0	nd	12	6	nd	0	0	GI	0		
15	0	59	3	30	5	3	3	0,27	0,5	0	50	50	50	1	1	Breast	1	1	116
16	0	23	22	32	1	0	0	0	0	0	12	12	12	1	1	Lungcancer	1		87
17	1	29	16	26	7	1	1	0,6	0	0	25	25	25	1	1	Prostata	1	1	66
18	0	27	10	29	5	3	4	0,17	0	0	12	12	12	1	1	Pancras	1		56

19	1	28	238	22	7	2	8	0,37	0	0	250	300	300	1	0	GI	1		
20	0	13	2	32	2	0	0	0	0	0	12	12	6	1	1	Gyn	1		103
21	1	112	40	33	5	3	7	0	0	0,27	12	25	50	1	0	Prostate	1	1	130
22	1	91	102	22	4	8	nd	0,3	0,73	nd	0	0	nd	0	0	Head-Neck	0		
23	0	21	4	26	5	2	7	0	0	0,23	0	0	0	1	0	Breast	1	1	86
24	0	62	20	34	5	4	5	0	0	0	0	0	0	1	1	GI	0		32
25	0	21	4	30	nd	nd	nd	0	0	0	0	0	0	1	1	Breast	1		55
26	1	10	14	25	7	7	5	0	0	0	25	0	0	1	0	Prostata	1	1	64
27	1	105	1	39	8	7	8	0	0	0	12	12	0	1	1	GI	0		83
28	0	41	208	26	5	5	nd	0	0	nd	0	0	nd	0	0	Gyn	1		
29	1	37	1	30	5	4	4	0	0	0	0	0	0	1	0	Brain tumor	0		
30	1	10	4	37	8	7	5	0	0	0	25	25	12	1	1	GI	1		30
31	0	52	13	39	1	0	nd	0	0	nd	0	0	nd	0	0	Pancreas	0		
32	1	38	1	28	5	5	6	0,3	0	0	0	0	0	1	0	Prostata	1	1	127
33	1	16	1	31	7	8	4	0,2	0	0	0	0	0	1	0	GI	1		46
34	1	8	1	30	3	2	3	0	0	0	25	0	12	1	0	GI	1		8
35	1	27	196	24	7	7	nd	0,2	0	nd	50	175	nd	0	0	Prostata	1	1	
36	0	26	1	34	8	7	6	0	0	0	25	50	50	1	1	Lungcancer	0		93
37	0	68	1	37	6	5	3	0,43	0	0	0	0	0	1	1	GI	1		68
38	1	12	32	24	4	6	nd	0	0	nd	25	0	nd	0	0	GI	0		
39	1	38	232	21	6	4	nd	0,73	0,1	nd	100	112	nd	0	0	Lungcancer	0		

Controls (untreated)	Sex 1=male	25-OHD nmol/L	CRP mg/L	Albumin g/L	**QoL** ESAS-score baseline	1 month	3 month	**Infections** percentage days with antibiotics baseline	1 month	3 month	**Pain** Phentanyl/hour baseline	1 month	3 month	**survival time** >3 months	> 6 months	Cancer	ongoing chemo.	ongoing anithorm. therapy	
1	1	58	18	30	nd	nd	nd	0	0	0,47	0	0	12	1	1	GI	0		
2	1	27	141	17	2	4	4	0	0	1	125	125	150	1	1	Pancreas	0		
3	0	39	42	31	0	0	nd	0,1	0,37		137	200	nd	0	0	Gyn	0		

4	0	33	ND	28	3	7	7	0	0,23	0,53	75	75	100	1	0	Breast	1	1
5	0	39	278	23	3	4	nd	0	0	nd	50	75	nd	0	0	Lungcancer	0	
6	0	20	83	18	4	4	8	0,67	0	0,47	25	150	250	1	1	Pancreas	0	
7	0	13	144	13	4	6	nd	0	0,3	nd	12	50	nd	0	0	Cholangiocarcino ma	0	
8	1	27	51	16	6	7	nd	0	0,4	nd	0	0	nd	0	0	Cholangiocarcino ma	0	
9	0	49	17	34	6	6	5	0	0,8	0,5	0	0	0	1	0	Head-Neck	1	
10	0	43	7	32	8	3	4	0,1	0,33	0	25	50	300	1	0	Breast	1	1
11	0	66	102	21	3	3	1	0	0,33	0,33	0	12	50	1	1	Breast	1	
12	0	47	24	29	2	0	nd	0	0,33	nd	37	75	nd	0	0	Lungcancer	0	
13	1	14	19	19	10	10	nd	0	0	nd	0	0	nd	0	0	GI	0	
14	0	8	23	30	1	1	nd	0,33	0	nd	112	250	nd	0	0	GI	0	
15	0	28	56	26	5	4	4	1	1	1	37	75	75	1	1	Breast	1	1
16	0	70	300	26	4	nd	0	0	0,5	0	0	0	0	1	1	Lungcancer	1	
17	1	20	9	22	3	3	5	0	0	0	12	12	12	1	1	Prostata	1	1
18	0	46	2	35	1	7	6	0	0	0	25	25	25	1	1	Pancras	1	
19	1	9	26	30	4	3	3	0	0	0,03	37	350	350	1	0	GI	1	
20	0	45	23	34	2	2	4	1	1	1	0	0	0	1	1	Gyn	1	
21	1	56	ND	33	6	6	7	0	0	0	50	75	100	1	0	Prostate	1	1
22	1	72	178	20	4	2	nd	0,27	0,2	nd	25	75	nd	0	0	Head-Neck	0	
23	0	33	19	30	8	0	5	0	0	0,03	25	50	100	1	0	Breast	1	1
24	0	86	43	32	4	4	7	0	0	1	0	225	300	1	1	GI	1	
25	0	65	5	36	4	1	2	0	0	0	50	150	125	1	1	Breast	1	
26	1	42	16	30	3	8	5	0	0	0	125	125	125	1	0	Prostata	1	1
27	1	35	7	37	1	0	0	0	0	0	50	50	37	1	1	GI	1	
28	0	11	15	34	10	8	nd	0,5	0,17	nd	87	150	nd	0	0	Gyn	0	
29	1	39	12	33	6	6	7	0	0	0,33	0	0	0	1	0	Brain tumor	0	
30	1	26	101	19	1	1	6	0	0,5	0,2	75	150	350	1	1	GI	1	
31	0	34	4	28	5	5	nd	0,63	0	nd	0	0	nd	0	0	Pancreas	0	
32	1	24	1	27	3	3	4	0	0	0	0	25	175	1	0	Prostata	1	1

33	1	41	ND	27	5	5	5	0	0	0,03	12	12	100	1	0	GI	0		
34	1	32	147	23	nd	nd	nd	0,33	0	0	37	125	150	1	0	GI	0		
35	1	43	143	26	0	6	nd	0	0,1	nd	50	50	nd	0	0	Prostata	1	1	
36	0	42	65	26	5	8	nd	0	0,13	0	50	50	75	1	1	Lungcancer	0		
37	0	32	137	11	4	2	0	0	0	0,57	0	25	75	1	1	GI	0		
38	1	48	1	29	7	8	nd	0,5	0	nd	100	300	nd	0	0	GI	0		
39	1	17	137	20	3	6	nd	0,23	0,1	nd	250	550	nd	0	0	Lungcancer	0		
